# Deficiency of caspase 3 in tumor xenograft impairs therapeutic effect of measles virus Edmoston strain

**DOI:** 10.18632/oncotarget.3496

**Published:** 2015-03-26

**Authors:** Biao Wang, Xu Yan, Qingguo Guo, Yan Li, Haiyan Zhang, JiSheng Xie, Xin Meng

**Affiliations:** ^1^ Department of Biochemistry and Molecular Biology, College of Basic Medical Sciences of China Medical University Shenyang, P.R. China; ^2^ Department of Prosthodontics, School of Stomatology, China Medical University, Shenyang, P.R. China; ^3^ Department of Oncology, Tumour Angiogenesis and Microenvironment Laboratory (TAML), First Affiliated Hospital, Liaoning Medical College, Jinzhou, P.R. China; ^4^ Department of Geriatrics, The First Affiliated Hospital, China Medical University, Shenyang, P.R. China; ^5^ Department of Ecsomatics, Youjiang Medical College for Nationalities, Baise City, P.R. China

**Keywords:** caspase-3, interferon alpha, oncolytic therapy, apoptosis, measles virus Edmonston strain

## Abstract

The oncolytic measles virus Edmonston (MV-Edm) strain shows considerable oncolytic activity against a variety of human tumors. In this study, we report MV-Edm is able to trigger apoptosis pathways in infected tumor cells and elucidate the roles of cellular apoptosis in the whole oncolytic process. We also show that activated caspase 3, a key executioner of apoptosis, plays key roles in the oncolytic virotherapy. Activated caspase 3 can accelerate viral replication in cervical cancer cells and enhance the killing effects of the virus. Deficiency of caspase 3 either in tumor cells or in tumor xenograft significantly desensitized tumor to oncolysis with MV-Edm. In the infected cells, caspase 3 regulates interferon α release, which can inhibit viral replication in neighboring tumor cells. We propose that caspase-3 activation enhances the oncolytic effects of MV-Edm, thus inhibiting tumor growth in mice.

## INTRODUCTION

Cervical cancer (CC) is one of the most common and lethal gynecological malignancies in both developing and developed countries [1, 2]. And oncovirotherapy is being touted as the future of cancer treatment, which has limited side effects and involves the use of the virus to specifically replicate in the tumor cells and kill the cells lytically. An oncolytic virus is a set of virus that preferentially infects and kills cancer cells rapidly and lytically [3–8]. Moreover, some of these viruses are being used as tools for cancer therapies in current clinical trials [9, 10], such as lymphoma, ovarian cancer, mesothelioma, breast cancer, and renal and hepatocellular carcinoma [9, 11–14].

Measles virus (MV) is a 16 kb single-stranded, negative-sense, enveloped RNA virus of the genus Morbillivirus within the family *Paramyxoviridae*. Many previous studies have shown that live attenuated Edmonston B strain of measles virus (MV-Edm) has potent and specific oncolytic activity against a variety of human cancer cells, including malignant lymphoma, multiple myeloma, mesothelioma, epithelial ovarian cancer, breast cancer, liver cancer and glioma [11, 15–19]. MV-Edm is selectively oncolytic, causing extensive syncytium formation and cell killing in a variety of human tumor cancer cells but minimal cytopathic damage in nontransformed cells such as normal skin fibroblasts, hepatocytes, mesothelial cells from the peritoneal cavity, and peripheral blood lymphocytes [11, 14, 19, 20].

As we know, both the pathogenic wild-type MV and the attenuated vaccine strain MV-Edm enter cells by binding through their H attachment protein to one of two cellular receptors, signaling lymphocyte activation molecule (SLAM, also known as CD150) or CD46 [21–23]. Actually, CD46 is frequently overexpressed on human cancer cells and barely expressed on normal cells (such as, fibrocytes and lymphocytes).

Human type I interferon (IFN-α/β) is crucial for host defense against viral infections. IFN-α/β have been proved to inhibit gene expression and the production of progeny virions of the measles virus vaccine strain, including Edmonston tag strain [24, 25]. It was reported that MV vaccine strains can induce significantly higher levels of type I IFN than wild-type MV [26].

Some studies have confirmed that oncolytic therapy is often accompanied by a process of inducing cell apoptosis [5, 27–29]. In our previous study, the whole process of oncolytic therapy for renal carcinoma was accompanied by apoptosis [14]. However, it still remains unclear whether the apoptosis induced by virus infection affects the virus replication and whether the cellular apoptosis could contribute to the oncolytic therapy.

As a key executioner in apoptosis, caspase 3 is considered an essential event during cellular apoptosis. We hypothesized that the cellular apoptosis could be involved in the process of cell death induced by MV-Edm infection and the caspase 3 mediated apoptosis could attenuate the innate immunity of host cells, thereby, accelerating the virus replication in cancer cells.

In this study, we evaluated the roles of caspase 3 mediated apoptosis in the oncovirotherapy in human CC cell lines and xenografts. Our data have demonstrated caspase 3 mediated apoptosis promoted the rapid replication of virus by inhibition of IFN production. We believe this newly discovered role of caspase-3 mediated apoptosis has profound implications for oncolytic cancer therapy.

## RESULTS

### CD46 is overexpressed on human CC cell lines

**Figure 1:**
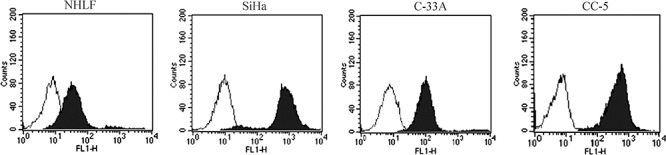
CD46 expression on the human cervical cancer (CC) cell lines SiHa and C-33A, primary human CC cells of CC-5, and normal human lung fibroblast cell line NHLF Relatively higher levels of CD46 receptor were observed on the human CC cell lines SiHa and C-33A, as well as the primary cancer cell CC-5 than that on normal cell line NHLF. The blank histograms show the measured fluorescence of cells incubated with an isotype control (detailed), and the black histograms represent cells labeled with an anti-CD46 fluorescein isothiocyanate antibody. The analysis was performed by flow cytometry.

CD46 expression in the normal human lung fibroblast cell line NHLF, human cervical cancer cell lines SiHa, C-33A and primary human cervical cancer cells of CC-5 was analyzed by flow cytometry. CD46 was expressed on the surface of all human cervical cancer cell lines and primary human cervical cancer cells: 97% in SiHa, 90% in C-33A, and 91–94% in CC-5 cells (*n* = 3). However, only 7% in NHLF demonstrated positive expression of CD46 (Figure 1). These results demonstrated that human cervical cancer cell lines and primary cervical cancer cells expressed higher levels of CD46 than normal cells.

### MV-Edm have a strong ability to induce cytopathic effects (CPEs) and cell death in CC cells

**Figure 2:**
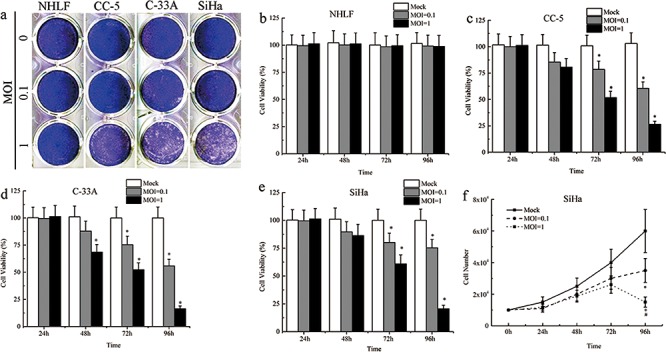
Cytopathic effects and cell death induced by MV-Edm **a.** Serial analysis to determine the cytopathic effects (CPEs) of MV-Edm was performed on the human CC cell lines SiHa and C-33A, primary cancer cells CC-5 and normal cell line NHLF. Ninety-six hours after infection at MOIs of 0.1 and 1, the cells were stained with crystal violet representing viable attached cells. **b–e.** The time course of cell viability of the human CC cell lines, primary cancer cells and normal cell line after infection with MV-Edm at MOIs of 0.1 and 1 was analyzed by the MTS assay. **f.** Live Cell counts assay was performed to evaluate the number of cells by Trypan Blue Staining. All data were presented as means ± SD. *means *P* < 0.05 when compared to MOCK group.

The cytopathic effects (CPEs) caused by MV-Edm replication were investigated in the normal human NHLF, SiHa, C-33A, and CC-5 cells. Cells were infected with MV-Edm at multiplicities of infection (MOIs) of 0.1 and 1 for 96 hours and then stained with crystal violet. MV-Edm infection caused dramatic CPEs in an MOI-dependent manner (*n* = 3; Figure 2a). However, normal human cell line NHLF showed minimal CPEs after MV-Edm infection (Figure 2a), even treated with MV-Edm at higher MOI. We further determined the cell viability after infection with the MV-Edm using the MTS Assay every 24 hours for 96 hours. The results showed that MV-Edm infection at MOI of 0.1 and 1 demonstrated a great cell growth inhibition in SiHa, C-33A, and primary CC-5 cells from 48 hours to 96 hours (*n* = 3; Figure 2b–2e). And MV-Edm at MOI of 1 has more inhibitory effects on the cell growth. Then, to confirm whether or not the cellular growth inhibition was caused by cell killing effects of MV-Edm, the cells were collected and counted with Trypen Bule staining method after infection with MV-Edm at different times. Briefly, SiHa, C-33A, and primary CC-5 cells were seeded at 1 × 10^4^ cells/well in a 6-well plate and incubated overnight. Then the cells were infected with MV-Edm at MOIs of 0.1 and 1, respectively. Collect all the cells every 24 hours and count the cells. At 72 h and 96 h, the cells-alive infected by MV-Edm were statistically lower in number than that in MOCK group (*P* < 0.05, *n* = 3; Figure 2e and Figure S-1). And at 96 h, cells in the MOI = 1 group was obviously lower in number than that in MOI = 0.1 group (*P* < 0.05, *n* = 3; Figure 2e–2f and Figure S-1).

### Role of Caspase-3 in the cellular apoptosis induced by MV-Edm infection *in vitro*

**Figure 3:**
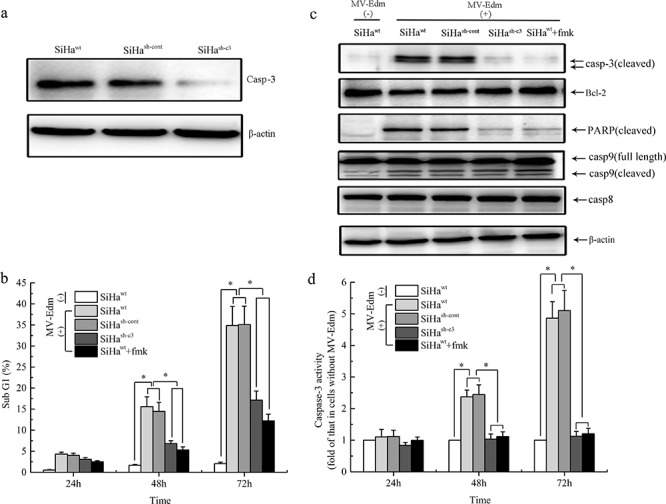
Role of Caspase-3 in the cellular apoptosis induced by MV-Edm infection *in vitro* **a.** SiHa cells were transfected with scramble shRNA or shRNA specific against Caspase-3 (sh-c3), and western blot analysis was performed using the indicated antibodies. **b.** The percentage of sub-G1 cells was measured by fluorescence-activated cell sorting. **c.** At 72 h, protein levels of Bcl-2, cleaved caspase-3 and PARP in the infected and uninfected SiHa cells were analyzed with Western Blot. **d.** At indicated times, the activity of caspase-3 from each group was determined using caspase-3 activity Kit. Data were presented as means ± SD. *means *P* < 0.05.

To investigate the function of caspase 3 in viral-infected CC cells, caspase 3 was down-regulated by short hairpin RNA (shRNA) against caspase 3 (sh-c3), which was confirmed by Western blot analysis (Figure 3a and Figure S-2a). The SiHa and C-33A cells were divided into four groups: the wild-type SiHa/C-33A cells without any treatments (SiHa^wt^/C-33A^wt^), SiHa/C-33A cells transfected with scrambled shRNA (SiHa^sh-cont^/C-33A^sh-cont^), SiHa/C-33A cells transfected with sh-c3 (SiHa^sh-c3^/C-33A^sh-c3^) and SiHa/C-33A cells treated with caspase 3 inhibitor Ac-DEVD-FMK (SiHa^wt^+fmk/C-33A+fmk). Each group was infected with MV-Edm at an MOI of 1, and apoptotic cells were analyzed by Sub G1 Assay and subsequent flow cytometry. Upon infection with the MV-Edm, the number of cells in sub-G1 increased in a time-dependent manner. In both SiHa^wt^ and SiHa^sh-cont^ groups, MV-Edm induced apoptosis in ~15% and ~35% of cells at 48 and 72 hours at an MOI of 1, respectively. However, at the same time points, MV-Edm only induced apoptosis in ~6% and ~17% of cells in both SiHa^sh-c3^ and SiHa^wt^+fmk groups (Figure 3b). The similar results were found in C-33A cell line (Figure S-2c). We further examined some protein expressions that were involved in cellular apoptosis, and found the MV-Edm increased the expressions of cleaved caspase 9, caspase 3 and cleaved PARP but decreasing the protein expression of Bcl-2 in the cells of SiHa^wt^ and SiHa^sh-cont^ groups, compared to those in SiHa^wt^ cells without virus infection, while caspase 8 was not activated. And compared to SiHa^wt^ and SiHa^sh-cont^ groups, the lower expressions of cleaved caspase 3 and cleaved PARP and higer Bcl-2 expression were obvious higher in cells of SiHa^sh-c3^ and SiHa^wt^+fmk groups (Figure 3c). The expressions of these key proteins were also determined in the C-33A cell line (Figure S-2b), which was similar with those results in SiHa cell line.

The caspase 3 activity was significantly increased after 48 and 72 hours of virus infection in SiHa^wt^ and SiHa^sh-cont^ groups while without any changes in cells of SiHa^sh-3^ and SiHa+fmk groups (Figure 3d), which was similar with the results of Western Blot. Also, the results were testified in the C-33A cell line (Figure S-2d).

### The role of caspase-3 in CC cell death induced by MV-Edm replication

**Figure 4:**
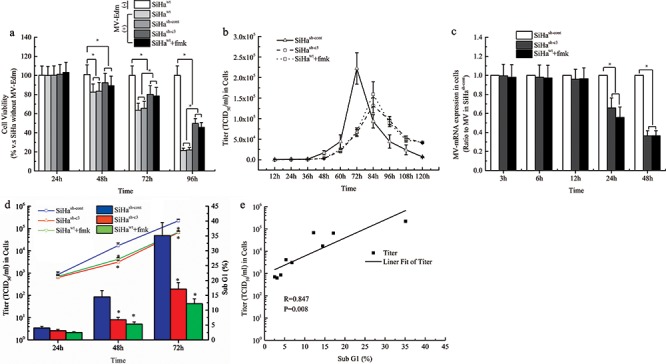
The role of Caspase-3 in the cell death induced by MV-Edm and the MV-Edm replication **a.** At indicated times, the cell viability of each group was assessed with MTS assay. **b.** The intracellular virus replication of SiHa cells, expressed as TCID_50_/ml, was determined at different times after infection at an MOI of 1. **c.** The viral mRNA levels were measured using real-time PCR. Each value is normalized to that in SiHa cells transfected with empty vector (SiHa^sh-cont^), which was set a ratio = 1, and represented with mean ± SD. **d.** The comparisons of virus production and cellular apoptosis induced by MV-Edm in SiHa cells from each group. **e.** Liner Fit curve between cellular apoptosis and virus production. *means *P* < 0.05.

To validate the role of apoptosis induced by caspase 3 in the oncolytic therapy with MV-Edm, the cell viability of each group was assessed with MTS method. The results showed that, after 72 and 96 hours of the virus infection, the cell viability in SiHa^wt^ and SiHa^sh-cont^ groups was obviously lower than that in SiHa^sh-c3^ and SiHa^wt^+fmk groups (Figure 4a). Then we determined the viral titers in cells infected with MV-Edm at an MOI of 1 from 12 to 120 hours with TCID_50_ method. We found that the viral titer in SiHa^sh-cont^ cells peaked at 72 h but for the viral titer in both SiHa^sh-c3^ and SiHa^wt^+fmk groups peaked at 84 h (Figure 4b). The viral replication was detected with qPCR and the result showed that the intracellular viral mRNA in SiHa^sh-cont^ cells was significantly higher than those in SiHa^sh-c3^ and SiHa^wt^+fmk groups at 24 h and 48 h after infection (*P* < 0.05, Figure 4c). After analysis on the data above, we found that the cellular apoptosis mediated by caspase 3 was positively correlated to the viral replication in a time dependent manner during the oncolytic process in CC cells (Figure 4d and 4e).

All the experiments have been performed and the corresponding results were confirmed in C-33A cell line (Figure S-3).

### Regulation of MV-Edm induced INF-α release and virus production by caspase 3

**Figure 5:**
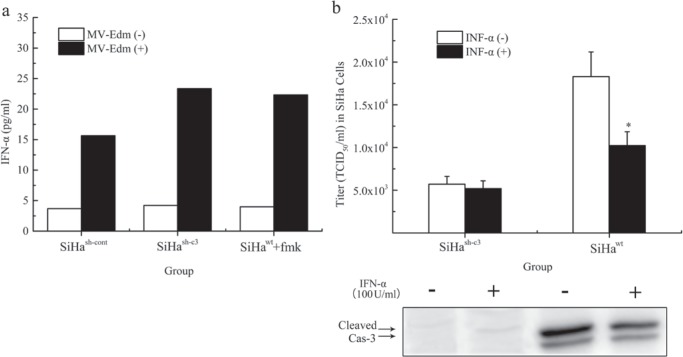
Regulation of MV-Edm induced INF-α release and virus production by caspase 3 **a.** After 48-hour infection, the production of IFN-α in SiHa cells was determined using human IFN-α enzyme-linked immunosorbent assay kit. The IFN-α data from samples were collected together in a single experiment. **b.** The intracellular virus replication and the expression of cleaved caspase 3 in SiHa cells co-treated with or without INF-α were compared at different 48 hours after infection at an MOI of 1. The data were presented as means ± SD. *means *P* < 0.05.

We next conducted experiments to confirm the role of caspase 3 in MV-Edm induced INF-α release and virus production. INF-α levels in SiHa^sh-cont^, SiHa^sh-c3^ and SiHa^wt^+fmk groups were determined with ELISA Kit at 48 hours after the viral infection (MOI = 1), respectively. We found that Caspase-3 inhibited INF-α release from infected cells (Figure 5a). To investigate whether IFN-α could prevent the virus replication through caspase 3, the cells were co-treated with Human IFN-α (100 IU/ml), and infected by MV-Edm at an MOI of 1. The intracellular titers, as well as the cleaved caspase 3, at different times after infection were determined respectively. Human IFN-α inhibited the viral replication and caspase 3 cleavage at 48 hours (Figure 5b).

The same experiments were carried out in C-33A cell line and the results were given in the Figure S-4.

### Deficiency in caspase 3 correlated with tumor response to oncolytic therapy in mice

**Figure 6:**
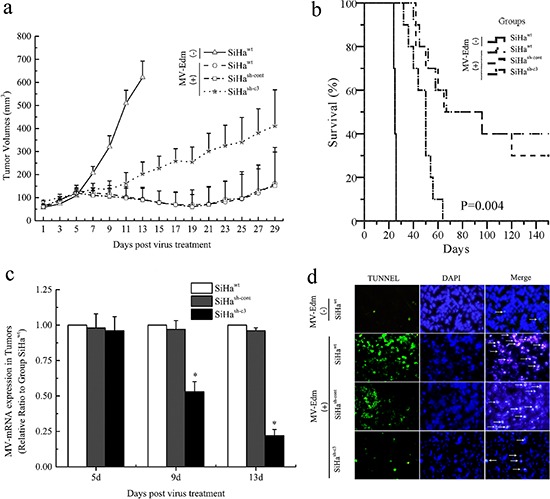
Deficiency in caspase 3 correlated with tumor response to oncolytic therapy in mice **a.** The increase in tumor volume after initiation of MV-Edm therapy. The data were presented as means ± SD. **b.** *Kaplan–Meier* survival curves of mice from different groups. **c.** The replication of MV-Edm in the xenografs was evaluated with qPCR. The data were presented as means ± SD. **d.** Morphological changes of apoptosis *in vivo* of SiHa cancer model. White arrows indicate apoptotic cells (TUNEL stain, × 200). *means *P* < 0.05.

To validate the role of caspase 3 mediated apoptosis on the oncolytic effects of MV-Edm *in vivo*, SiHa^wt^, SiHa^sh-cont^, and SiHa^sh-c3^ cells were injected s.c. into the right (or left) flank of BALB/c-nu/nu mice. All animals were examined daily for the appearance of palpable tumors. When the tumors were more than 5 mm in diameter at the time of measurement, MV-Edm was given intratumorally to nude mice bearing established subcutaneous xenografts (*n* = 10). Intratumoral administration of MV-Edm (10 doses of 1.0 × 10^6^ TCID_50_/dose) effectively suppressed the SiHa^wt^ and SiHa^sh-cont^ xenografts than SiHa^sh-c3^ xenografts (Figure 6a). At 150 days after injection, the survival rate was significantly improved in the SiHa^wt^ and SiHa^sh-cont^ groups (30–40%), compared to the SiHa^sh-c3^ group (0%) (Figure 6b). The xenografts infected with MV-Edm had mRNA expression of the M gene of MV-Edm and the results showed that MV-Edm replicated in the SiHa^wt^ and SiHa^sh-cont^ xenografts, but less effectively in SiHa^sh-c3^ group, at 5, 9 and 13 days after the viral injection (*P* < 0.05) (Figure 6c). And the apoptosis in the xenografts of each group was confirmed by TUNEL assay on the 13^rd^ day of virus treatment (Figure 6d).

## DISCUSSION

Oncolytic virotherapy is an emerging treatment modality that uses replication-competent viruses to destroy cancers specifically, which are currently being explored in the clinic [30–35]. And oncolytic viruses are genetically programmed to replicate within cancer cells and directly induce toxic effect via cell lysis or apoptosis.

In clinical trials, it was reported that live-attenuated MV strain has been used as an option of treatment for patients with stage I or II cancer [36]. Although MV treatment was tested in many kinds of tumor cells, MV has not been reported in the treatment of CC as a common virus system for oncolytic therapy. And besides this, that oncolytic therapy compounds with cellular apoptosis is not well elucidated at present. Here, for the first time, we described cellular apoptosis induced by infection of MV-Edm was mainly mediated by caspase 3 and elucidated the role of the caspase 3 mediated apoptosis in the process of oncolytic virotherapy with MV-Edm, besides confirming the potent antitumor activity of MV-Edm against human CC cells *in vitro* and *in vivo*.

It has been reported that the effectiveness of MV-Edm-mediated oncolysis is dependent upon the expression of the cellular attachment receptor CD46 which is more frequently over-expressed in human cancer cells than in normal cells. Our data demonstrated that CD46 was overexpressed in human CC cell lines as well as primarily cultured human CC cells with 11-fold higher than in the normal human NHLF cell line. That made it possible for the MV-Edm to infect human CC cells by preferentially binding to CD46.

Compared to cancer cells, MV-Edm induces minimal CPEs in normal human cell line, suggesting that CC cancer cells were suitable targets for MV-Edm infection. In our experiments, we found that MV-Edm at an MOI of 1 killed the human CC cells more effectively, which was validated by cell counting, while virus at an MOI of 0.1 only inhibited the cell growth. We further determined the replication ability of MV-Edm in the CC cells using the Tissue Culture Infectious Dose 50% (TCID_50_) Assay every 12 hour for 120 hours. We found that MV-Edm strains had a strong ability to replicate in CC cells in a time-dependent manner. The results of cell counting and viral titer changes suggested that the cell growth was inhibited at the early-stage of infection CC cells, then the cells was killed and lysed at the late-stage of viral infection. At the same time, the viral replication was also proved to be time-dependent through detection of the viral M gene [14]. Therefore, we esteemed that the oncolysis in CC cells was induced by MV-Edm mRNA transcription followed by abundant intracellular virus production which accelerated cancer cells lysis and led to cellular necrosis after viral infection.

Apoptosis is often called programmed cell death. Now, a large number of studies have shown that chemotherapy and radiotherapy can induce apoptosis in tumor cells [37, 38]. However, apoptosis is a double-edged sword. It is reported that drug-induced apoptosis can enhance oncolytic effect of MV-Edm in glioblastoma [39], but apoptosis via activation of caspase-3 during radiotherapy can mediate stimulation of tumor repopulation [40]. However, the role of caspase 3 in the oncolytic therapy with MV-Edm remains unclear. Our previous data have shown that our engineered MV had the ability to rapidly induce apoptosis in human renal cancer cells [14]. In this study, we found that MV-Edm also effectively induced the apoptosis in CC cells in a time- and caspase-3 dependent manner. The cell viability of caspase 3 deficient cells was obviously higher than that in wild-type cells after viral infection, and knockdown of caspase 3 attenuated the ability of MV-Edm to induce the apoptosis and reduce viral replication capacity in CC cells, suggesting that caspase 3 played a vital role during the process of the oncolysis.

Interferon α (IFN-α) is an important factor in the innate immunity and is essential for the response to measles virus [26, 41]. It acts on neighboring cells that are not apoptotic yet. In paracrine manner, in these neighboring cells, IFN-a decrease viral replication [42]. In most cases, to combat the cellular innate immune response, many viruses encode antagonistic proteins that block some steps of the IFN-α antiviral response. We proved that the cellular apoptosis mediated by caspase 3 inhibited the inner IFN α release after 48 hours of the infection, which resulted in the loss of natural barriers to virus replication in the cells. Our further findings showed that IFN α at a relatively low concentration could not effectively suppress the viral replication in caspase 3 deficient CC cells, while it functioned effectively in the wild-type cells. And the expression of cleaved caspase 3 was also decreased in the IFN α treated cells after the viral infection, suggesting that IFN α not only inhibited the virus replication directly but also functioned through the down-regulation of cleaved caspase 3 in CC cells, which has been proved by the results of virus titer with TCID_50_ method. In animal experiments, we found that caspase 3 mediated apoptosis enhanced the oncolytic effects of MV-Edm in CC tumor, which coincided with the results from those results *in vitro*.

In conclusion, MV-Edm has potent therapeutic efficacy in human CC both *in vivo* and *in vitro*. During the process of MV-Edm infection, MV-Edm could escape from the host immune surveillance, at least partially, through activing caspase 3 to inhibit IFN α release in CC cells. We believe caspase 3 can be a new target to enhance the oncolytic effects of MV-Edm in CC cells. That whether oncolytic therapy with MV-Edm can be enhanced via chemoradiation mediated apoptosis is our research focus in the future.

## MATERIALS AND METHODS

### Cell culture

The human CC cell lines SiHa and C-33A (ATCC, Manassas, VA) were maintained in Eagle's Minimum Essential Medium supplemented with 10% heat-inactivated fetal bovine serum (Bioserum, Japan). The normal human lung fibroblast cell line NHLF (Clonetics, San Diego, CA) were maintained in Dulbecco's modified Eagle's medium supplemented with 10% heat-inactivated fetal bovine serum (Bioserum, Japan). Vero cells were used to produce measles virus and maintained in Dulbecco's modified Eagle's medium supplemented with 5% heat-inactivated fetal bovine serum (Bioserum, Japan). All media used in this study contained 100 U/ml of penicillin - streptomycin. Primary human CC tissues were established using surgical specimens immediately after resection from the First Affiliated Hospital of China Medical University after institutional review board approval and informed patient consent. Briefly, tissues were treated with collagenase (GIBCO, Invitrogen, Carlsbad, CA) at 37°C for 2 hours on a shaker, and then filtered through a nylon mesh (100-μm diameter) to obtain single cell suspensions. Harvested cells were cultured in Minimum Essential Medium-α medium (GIBCO, Invitrogen) supplemented with 10% fetal bovine serum (Hyclone, Logan, UT) and 4 μg/ml of Gentamicin Reagent Solution (GIBCO, Invitrogen). All cell lines used in this study were cultured in a humidified atmosphere containing 5% CO_2_ at 37°C.

### Flow cytometry

CD46 expression and the apoptosis of cells were determined by flow cytometry. To measure CD46 expression, the cells were harvested and incubated with a fluorescein isothiocyanate–labeled monoclonal mouse antihuman CD46 or control antibodies (BD Biosciences, Pharmingen) for 0.5 hour on ice. Then cells were washed twice with cold PBS and 1 × 10^4^ cells per sample were analyzed using a FACScan (BD Biosciences, San Jose, CA). For sub-G1 analysis, SiHa cells were seeded in 6-well plates and infected with MV-Edm at an MOI of 1. All the cells were harvested at 24, 48, and 72 hours after infection and fixed in ice-cold 70% ethanol for at least 1 hour. Then the cells were rinsed twice with PBS and incubated for 30 minutes at room temperature in 1 ml PBS containing 50 μg propidium iodide (Sigma-Aldrich, St Louis, MO), 0.1% Triton X-100, 1 mmol/l EDTA, and 0.5 mg RNaseA. The stained cells were subjected to flow cytometric analysis with a FACScan (BD Biosciences, San Jose, CA). Fragmented, apoptotic nuclei were recognized by their sub-G1 DNA content. The proportion of cells in sub-G1 phase was determined for each group.

### Evaluation of CPEs *in vitro*

SiHa, C-33A, CC-5 (primary cultured human CC cells), and NHLF cells were cultured in 24-well plates at a density of 2 × 10^4^ cells/well. The cells were infected with MV-Edm at an MOI of 1 or 0.1 in 0.2 ml of Opti-MEM I (GIBCO, Invitrogen) for 2 hours. The virus suspension was removed, and 1 ml of fresh medium was added to each well. At 120 hours after infection, the cells were gently washed twice with PBS, and the remaining cells were fixed with 0.5% glutaraldehyde in PBS for 15 minutes. Then, cells were washed with PBS and stained with 0.1% crystal violet solubilized in 2% ethanol–distilled water. The stained product was subsequently washed twice with distilled water, air-dried, and then photographed.

### Cell counting with trypan blue staining

To determine cell viability and viable cell yield with the Trypan Blue Exclusion Method, follow the directions below. SiHa cells were cultured in 24-well plates at a density of 2 × 10^4^ cells/well and infected with MV-Edm at an MOI of 1. After 24 h, 48 h, 72 h and 96 h, prepare the cells suspension in Hanks' Balanced Salts Solution (HBSS) and then transfer 0.5 ml of 0.4% Trypan Blue solution (w/v) to a test tube. Add 0.3 ml of HBSS and 0.2 ml of the cell suspension (dilution factor = 5) and mix thoroughly. Allow to stand for 5 to 15 minutes.

### Stable shRNA mediated repression of caspase 3 in SiHa cells

The expression of human caspase 3 in SiHa and C-33A cells was silenced by shRNA interference. The shRNA against caspase 3 (NM_004346) system was designed and purchased from GeneChem Corporation (Shanghai, China). An adopted non-silencing control shRNA sequence that was not complementary to any human gene was used as a control shRNA. The sequences of these shRNA is: 5′-CCGGGCGAATCAATGGACTCTGGAA CTCGAG TTCCAGAGTCCATTGATTCGC TTTTTG-3′ (in bold red are the sense and antisense targeting sequences and in underlined blue is loop sequence). SiHa and C-33A cells were transfected with Lipofectamine 2000 reagent (Invitrogen) as instructed by the supplier and subjected to western blot to test the expression level of caspase 3 in the transfected cells.

### Western blot analysis

The cells were scraped in cold PBS and lysed in buffer containing 50mM Tris pH 7.8, 150 mM NaCl, 5 mM EDTA, 1 mM Na_3_VO_4_, 10 mM NaF, 10 mM NaPyrophosphate, 1% NP-40 and 1/7 of Protease cocktail inhibitors (Roche). Western blotting was done using standard 10% SDS-PAGE gel, loading 40 μg of protein per lane, with detection by enhanced chemiluminescence. For apoptosis assessment, antibodies against caspase-3 (dilution 1:1000, Cell Signaling Technology), Bcl-2 and Bax (dilution 1:1000, Cell Signaling Technology) were used. For loading control we used β-actin (dilution 1:5000, Cell Signaling Technology). All the primary antibodies were incubated for 2 hours at RT. The immune-reactivity of the blots was visualized using an enhanced chemiluminescence detection system (Amersham, Piscataway, NJ).

### Cell proliferation assay

The Cell-Titer 96 Aqueous Non-Radioactive Cell Proliferation Assay (Promega, Madison, WI) was used in this study. SiHa, C-33A, CC-5, and NHLF cells were seeded in 96-well plates at a density of 1 × 10^4^ cells/well. Twelve hours after seeding, the cells were infected with MV-Edm at an MOI of 1 for different time intervals and then incubated with 20 μl of MTS reagent for 4 hours at 37°C. The absorbance at 490 nm was recorded using an ELISA plate reader.

### Real time RT-PCR

The cells or the xenograft tissues were collected at different time intervals. Total RNA was extracted with miRNeasy/Protect Mini Kit (QIAGEN, Shanghai, China) according to manufacturer's instructions. Primers for M gene were designed mainly using Primer Premier 6 software. The following primers were used for qRT-PCR by SYBR Green method: 5′-GTTATGGACTCGCTATCTGT-3′ (Sense primer) and 5′-CGGTGCTTGATGTTCTGA-3′ (Antisense primer). Real-time PCR was performed on ABI 7500 PCR Instrument (ABI, Foster City, CA, USA) with a SYBR Green Real-time PCR Master Mix Kit (Toyobo, Tokyo, Japan). The relative mRNA levels of MV-Edm in cells were calculated using the 2 ^− ßßCT^ method with the endogenous β-actin mRNA as control.

### Assessment of MV replication in a human CC cells

The human CC cell lines were seeded in 6-well plates at a density of 2.0 × 10^4^ cells/well. Twelve hours after plating, the cells were infected with MV-Edm at an MOI of 1 in Opti-MEM I. The cells and supernatants were collected at different time intervals. The viruses were released by two cycles of freezing and thawing. The viral titers in the cells and supernatants were determined by titrating the TCID_50_ on Vero cells.

### *In vivo* xenograft experiments

Athymic female nude mice (BALB/c-nu/nu, 4 weeks old, and weighing~20g; Vital River Laboratories, Inc., Beijing, China) were injected s.c. into the right (or left) flank with 2 × 10^6^ SiHa cells in 100 μl PBS. After tumors were established, nude mice with SiHa tumors were randomly assigned to two groups with 10 mice in each group. MV-Edm group: the mice were injected with treated with MV-Edm (2 × 10^5^ TCID50 in 50 μl Opti-MEM I) every 2 days, total 10 times; MOCK group: the mice were treated with Opti-MEM I containing no virus, correspondingly. Tumors were measured every 2 days and tumor sizes were calculated by using the function [a × 0.5 b^2^], where a and b are the length and width of tumors, respectively.

Mice were killed if they lost > 20% of their body weight or the tumor diameter exceeded 1.0 cm. All mouse experiments were approved by the Committee of the Ethics on Animal Experiments in the Faculty of Medicine, China Medical University and carried out following the Guidelines for Animal Experiments in the Faculty of Medicine, China Medical University and The Law and Notification of the Government.

### *In situ* cell death (apoptosis) detection by TUNEL labeling

*In situ* hybridization for terminal deoxynucleotidyl transferase-mediated nick end labeling (TUNEL) was performed on paraffin sections as recommended by the manufacturer (Merck Millipore, Kilsyth, Vic, Australia). Apoptotic cells were identified by double labelling using TUNEL. And sections were counterstained with DAPI. These glass coverslips were then visualized under a fluorescence microscope (OLYMPUS TH4-200).

### Statistical analysis

Each experiment was repeated three different times, and data are presented as means ± SD. The data were analyzed by a one-way analysis of variance (ANOVA) or unpaired Student's *t* test with Bonferroni correction. Statistical analysis of tumor volumes before and after infection among the groups was performed using the Kruskal–Wallis test. Wilcoxon Rank-Sum test was used to determine two-group tumor volume comparisons. To assess survival, Kaplan–Meir curves were generated. The survival of mice in the different treatment groups was compared using the log-rank test. All analysis was performed using SPSS 13.0 software (SPSS, Chicago, IL). A *p* value of < 0.05 was considered statistically significant.

## SUPPLEMENTARY FIGURES


