# Supplementary material for: Deficiency of caspase 3 in tumor xenograft impairs therapeutic effect of measles virus Edmoston strain
Source: Oncotarget. 2015 Mar 26;6(18):16019–30. doi: 10.18632/oncotarget.3496 (PMC4599253; doi:10.18632/oncotarget.3496)
Supplement: Supplementary file 1 [file oncotarget-06-16019-s001.pdf]

SUPPLEMENTARY FIGURES

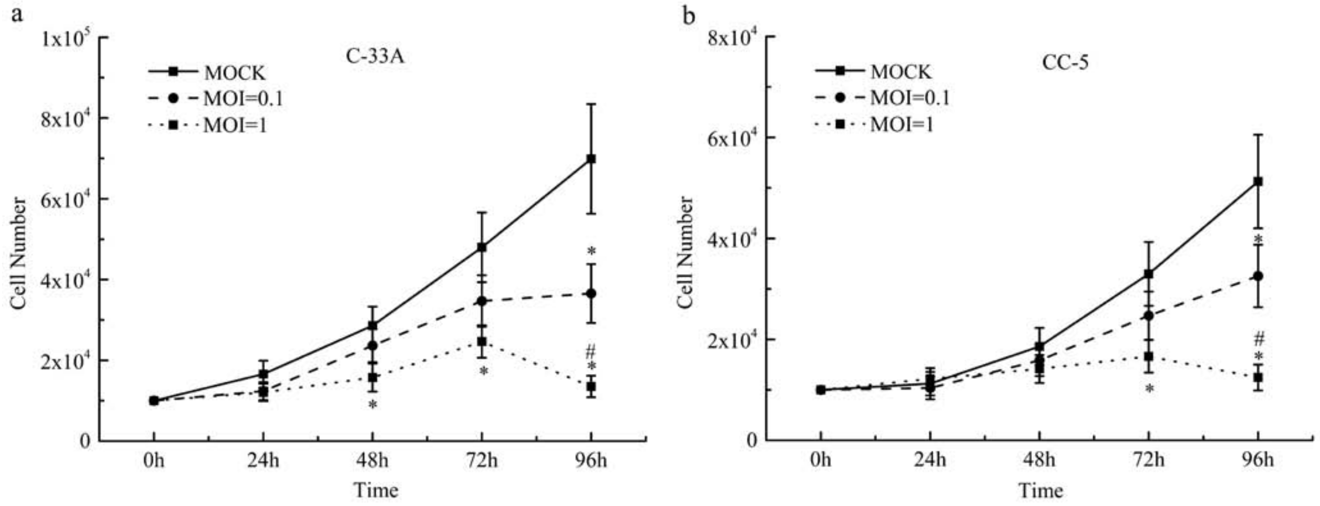

Supplementary Figure S1: Cell Counts *in Vitro*. Cell counts assay was performed to evaluate the number of cells by Trypan Blue Staining. All data were presented as means ± SD. *means $P < 0.05$ when compared to MOCK group.

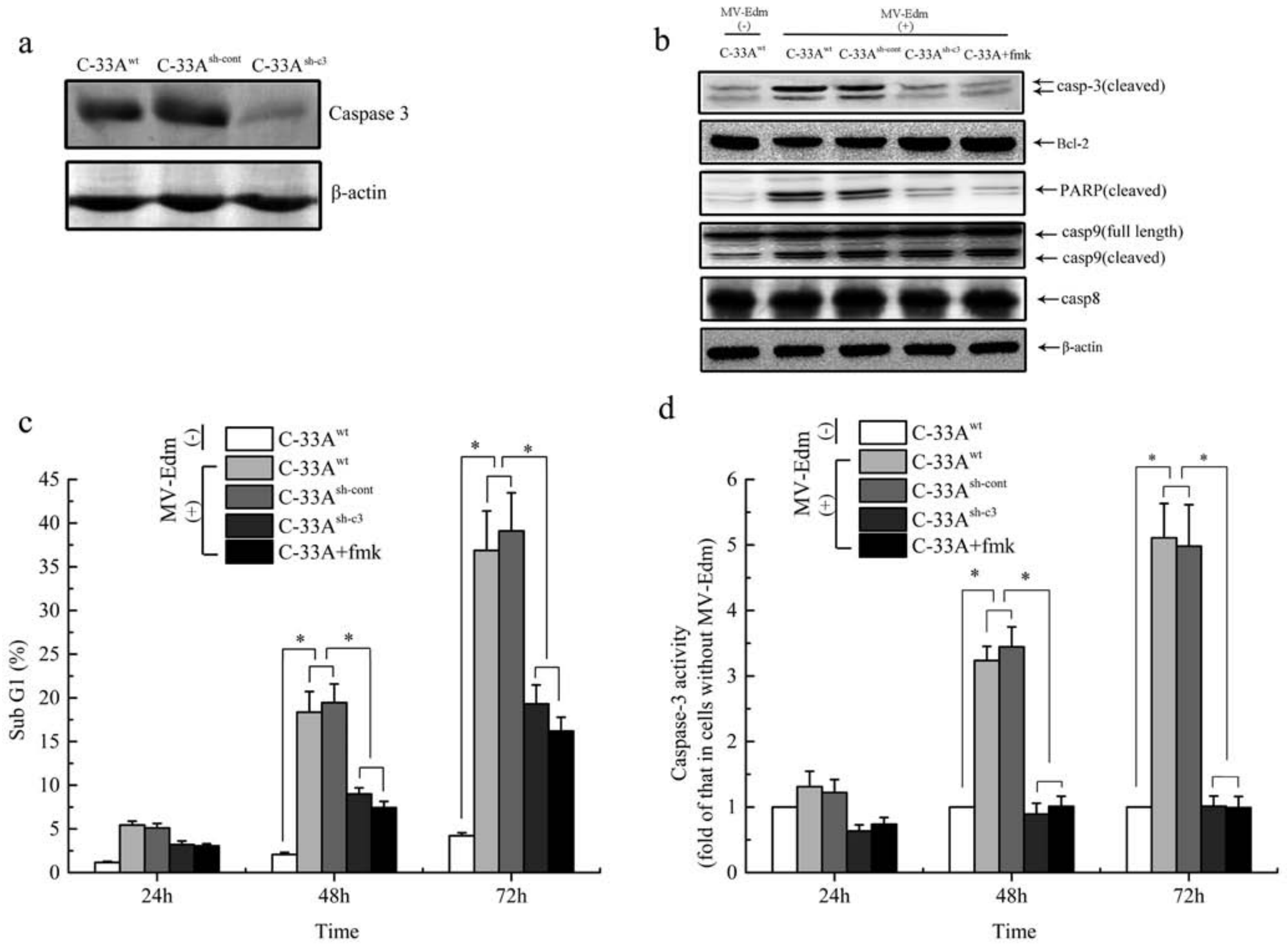

Supplementary Figure S2: Role of Caspase-3 in the cellular apoptosis induced by MV-Edm infection in C-33A cells. a. C-33A cells were transfected with scramble shRNA or shRNA specific against Caspase-3 (sh-c3), and western blot analysis was performed using the indicated antibodies. **b.** At 72 h, protein levels of Bcl-2, cleaved caspase-3 and PARP in the infected and uninfected C-33A cells were analyzed with Western Blot. **c.** The percentage of sub-G1 cells was measured by fluorescence-activated cell sorting. **d.** At indicated times, the activity of caspase-3 from each group was determined using caspase-3 activity Kit. Data were presented as means ± SD. *means $P < 0.05$.

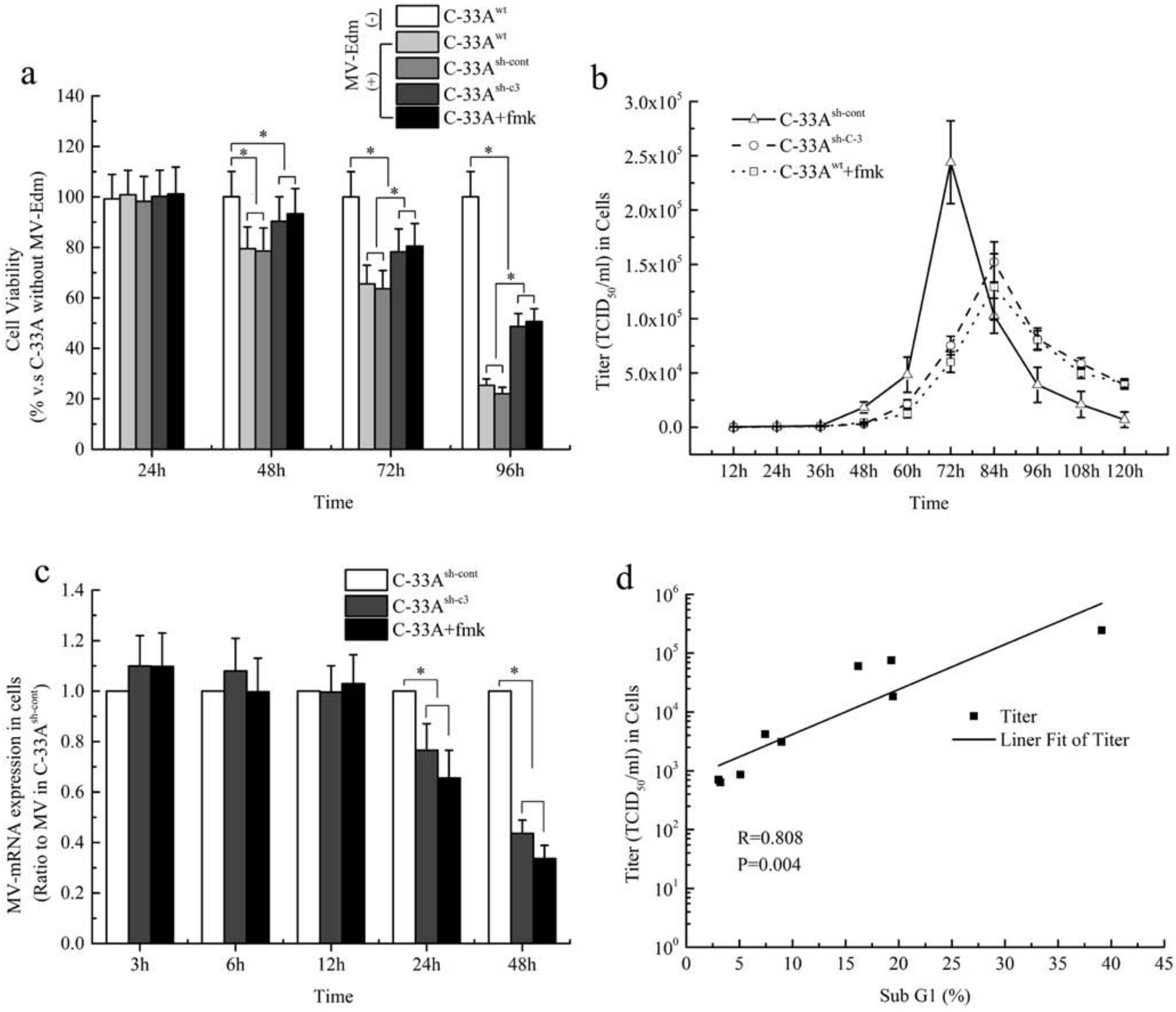

Supplementary Figure S3: Role of Caspase-3 in the cell death induced by MV-Edm and the MV-Edm replication in C-33A cells. a. At indicated times, the cell viability of each group was assessed with MTS assay. **b.** the intracellular virus replication of C-33A cells, expressed as $TCID_{50}$/ml, was determined at different times after infection at an MOI of 1. **c.** The viral mRNA levels were measured using real-time PCR. Each value is normalized to that in C-33A cells transfected with empty vector (C-33A$^{sh\text{-}cont}$), which was set a ratio = 1, and represented with mean ± SD. **d.** Liner Fit curve between cellular apoptosis and virus production. *means $P < 0.05$.

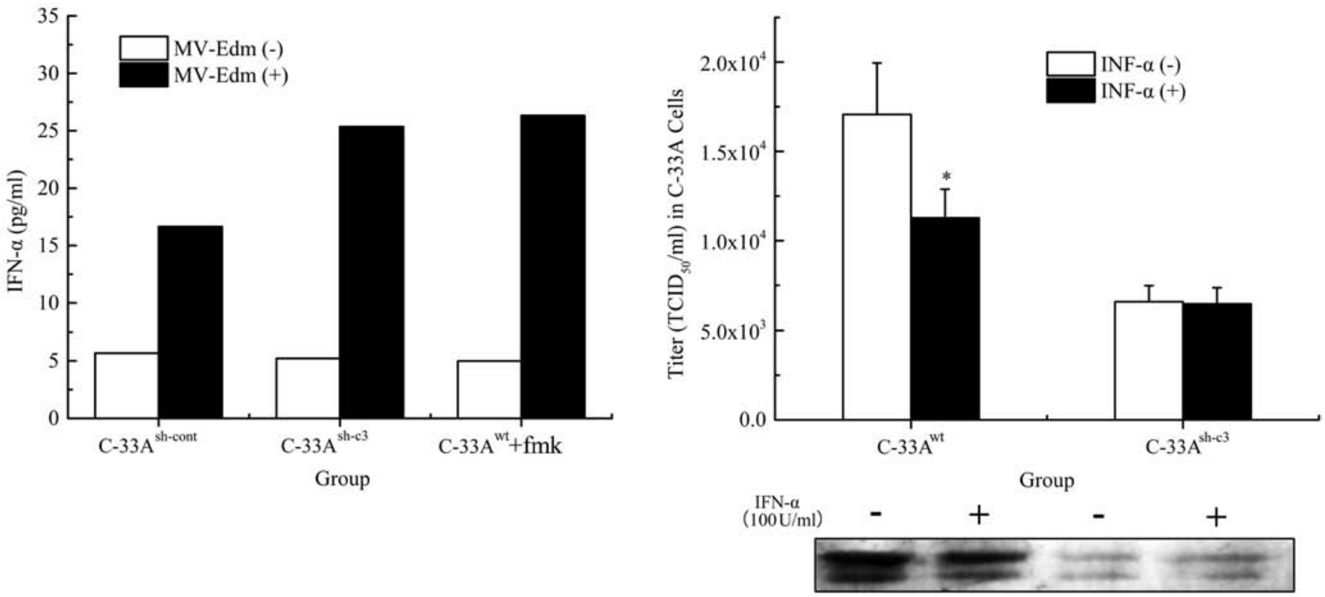

Supplementary Figure S4: Regulation of MV-Edm induced INF-α release and virus production by caspase 3 in C-33A cells. a. After 48-hour infection, the production of IFN-α in C-33A cells was determined using human IFN-α enzyme-linked immunosorbent assay kit. **b.** The intracellular virus replication and the expression of cleaved caspase 3 in C-33A cells co-treated with or without INF-α were compared at different 48 hours after infection at an MOI of 1. Data were presented as means ± SD. *means $P < 0.05$.
